# Multidimensional assessment of infant, parent and staff outcomes during a family centered care enhancement project in a tertiary neonatal intensive care unit: study protocol of a longitudinal cohort study

**DOI:** 10.1186/s12887-023-04165-0

**Published:** 2023-07-07

**Authors:** Rahel Schuler, Lea Woitschitzky, Carola Eiben, Judith Beck, Alena Jägers, Anita Windhorst, Birgit Kampschulte, Jutta Petzinger, Markus Waitz, Monique Oude Reimer-van Kilsdonk, Bernd A. Neubauer, Klaus-Peter Zimmer, Harald Ehrhardt, Burkhard Brosig, Walter A. Mihatsch

**Affiliations:** 1 Department of General Pediatrics and Neonatology, Justus- Liebig- University, Feulgenstrasse 12, 35392 Giessen, Germany; 2 Department of Psychosomatic Medicine, Justus-Liebig-University, Feulgenstrasse 12, 35392 Giessen, Germany; 3 Institute of Medical Informatics, Justus -Liebig -University, 35392 Giessen, Germany; 4 Department of Neonatology, Erasmus Medical Centre, Rotterdam, Netherlands; 5 Department of Neuropediatrics, Justus- Liebig- University, Feulgenstrasse 12, 35392 Giessen, Germany; 6 Department of Pediatrics, University of Ulm, Ulm, Germany; 7 University of Applied Sciences, Neu Ulm, Germany

**Keywords:** Family centred care, Family integrated care, Preterm infants, Discharge, Depression in mothers and fathers, Staff satisfaction, NICU, PDSA

## Abstract

**Background:**

The therapeutic advances and progress in the care for preterm infants have enabled the regular survival of very immature infants. However, the high burden of lifelong sequelae following premature delivery constitutes an ongoing challenge. Regardless of premature delivery, parental mental health and a healthy parent–child relationship were identified as essential prerogatives for normal infant development. Family centered care (FCC) supports preterm infants and their families by respecting the particular developmental, social and emotional needs in the Neonatal Intensive Care Unit. Due to the large variations in concepts and goals of different FCC initiatives, scientific data on the benefits of FCC for the infant and family outcome are sparse and its effects on the clinical team need to be elaborated.

**Methods:**

This prospective single centre longitudinal cohort study enrols preterm infants ≤ 32 + 0 weeks of gestation and/or birthweight ≤ 1500 g and their parents at the neonatal department of the Giessen University Hospital, Giessen, Germany. Following a baseline period, the rollout of additional FCC elements is executed following a stepwise 6-months approach that covers the NICU environment, staff training, parental education and psychosocial support for parents. Recruitment is scheduled over a 5.5. year period from October 2020 to March 2026. The primary outcome is corrected gestational age at discharge. Secondary infant outcomes include neonatal morbidities, growth, and psychomotor development up to 24 months. Parental outcome measures are directed towards parental skills and satisfaction, parent-infant-interaction and mental health. Staff issues are elaborated with particular focus on the item workplace satisfaction. Quality improvement steps are monitored using the Plan- Do- Study- Act cycle method and outcome measures cover the infant, the parents and the medical team. The parallel data collection enables to study the interrelation between these three important areas of research. Sample size calculation was based on the primary outcome.

**Discussion:**

It is scientifically impossible to allocate improvements in outcome measures to individual enhancement steps of FCC that constitutes a continuous change in NICU culture and attitudes covering diverse areas of change. Therefore, our trial is designed to allocate childhood, parental and staff outcome measures during the stepwise changes introduced by a FCC intervention program.

**Trial registration:**

Clinicaltrials.gov, trial registration number NCT05286983, date of registration 03/18/2022, retrospectively registered, http://clinicaltrials.gov.

## Background

Family centred care (FCC) acknowledges that emotional, social and developmental support are integral components of health care. The respect for the infants’ and the families’ innate strengths are at the core of FCC as well as supporting families in their caregiving and decision-making roles [1]. In 1993 Helen Harrison introduced the principles of FCC in the Neonatal Intensive Care Unit (NICU) [2]. Since that time various FCC initiatives have been implemented, although inconsistently, in NICUs all over the world [3–5].

FCC is more of a concept that needs to be integrated into the culture of the NICU and the attitude of each individual than a set of tasks to be accomplished [6, 7] At the core of FCC is providing healthcare in the context of the strengths and needs of the patients, their family and the community. The goal is to improve quality, psychological wellbeing, clinical outcomes and the overall patient and family experience [6].

Technical and medical advances greatly improved the outcome of preterm infants, especially for the most immature, [8] but NICUs are highly professionalized and often stressful environments for preterm infants and their parents [9]. In spite of these advances neonatal morbidities (e.g. bronchopulmonary dysplasia, retinopathy of prematurity, severe brain injury) continue to affect premature infants and they still have more neurobehavioral problems and a poorer neurodevelopmental outcome than their term counterparts [10, 11]. The stressful NICU environment and the stressful experience of the parents might further aggravate these undesirable outcomes. FCC has been introduced into the care of preterm infants to reduce stress for the infant and the whole family.

FCC interventions and outcomes vary significantly across different studies [12]. Parents were involved in the care to different degrees, ranging from interventions to support parents, interventions that are delivered by parents to concepts that focus on partnering with parents as equal counterparts in the care of their infant [6]. In a recent review FCC interventions improved weight gain, readmission rates and parental satisfaction, however, length of stay and neonatal morbidities were not improved [12]. Family integrated care (FiCare) has been developed in Canada and is currently the FCC programme that has been tested in the largest group of preterm infants with improved infant weight gain and motor development and reduced parental anxiety [12–14]. FiCare is built on four pillars: Parent education, staff education, NICU environment and psychosocial support: [13].

The present study will focus on interventions following the 4 pillar concept.

The primary outcome of the study is length of hospital stay measured by corrected gestational age at discharge. We hypothesize that empowerment of parents and their increasingly active involvement in infant care will reduce length of hospital stay, as parents will be ready to take their infant home earlier [15].

**Fig. 1:**
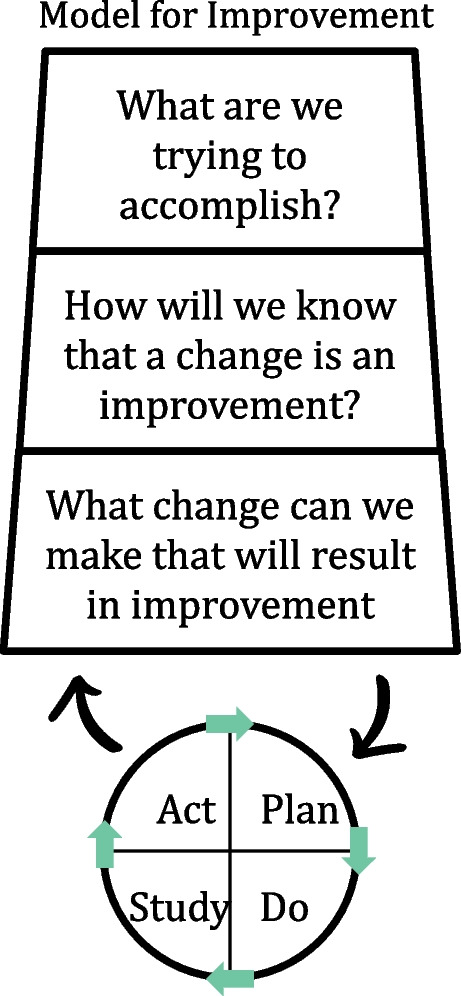
Model for improvement

Quality improvement (QI) methods have become a central part of neonatology within the last years. One widely used approach is the Model for Improvement and the PDSA (Plan- Do- Study- Act) cycle (Model for Improvement, Fig. 1) [16].

The Model of Improvement asks three questions: What are we trying to accomplish? (aim) How will we know that a change is an improvement? (measurement) What change can we make that will result in improvement? (wise guess) [16].

**Fig. 2:**
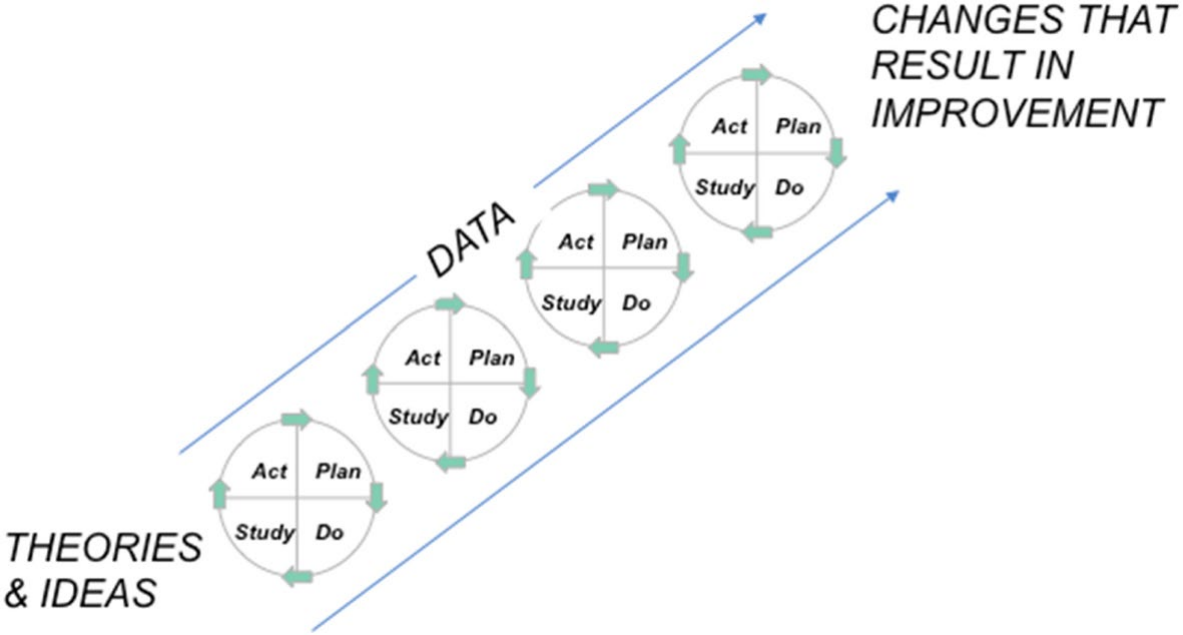
PDSA cycles

The PDSA cycle is a continuous circuit of sequential cycles with the complexity and size of cycles increasing with every cycle [17]. The cycle starts with a change concept (Plan), the next step is the implementation of the new intervention (Do), followed by qualitative and quantitative monitoring and data collection (Study) and the final step of integrating the learning generated which will then lead to acceptance, rejection or adaptation of the intervention (Act), [16] followed by the next PDSA cycle (PDSA cycles, Fig. 2).

Although widely used in healthcare two reviews of 2014 and 2019 have shown that the five key principles of the PDSA cycle are applied inconsistently with only 3% and 4% of the reviewed studies applying all of the following key principles: sufficient documentation of PDSA cycles, iterative cycles, small-scale-testing, continuous data collection and theoretical rationale [17, 18]. Consistent improvements require iterative adaptation to the local context therefore greater scientific rigour is needed in the application and reporting of these methods [18].

Despite the challenges in its most effective application, PDSA cycles have been used successfully [19–21]. The strength of the PDSA cycle is the iterative approach to test and assess an intervention rapidly and make necessary changes timely [18]. The present study will use the Model for Improvement and the PDSA cycle to improve FCC in our level III NICU.

An urgent need is seen to standardize FCC interventions and core outcome measures to enable future comparison of the efficiency of FCC interventions and thus to improve the evidence base [12]. These outcome measures need to involve the infant, the parents and the medical team, as all are closely linked and interdependent.

Therefore, a comprehensive standardized assessment tool assessing the advances of parent, infant and staff outcome in the context of the degree of FCC implementation was developed. The aim of the present study is to test this new assessment tool in a prospective single centre longitudinal cohort study.

## Methods/design

The study design is a prospective single centre longitudinal cohort study as the enhancement of FCC primarily is a change in unit culture and the attitudes of individuals [7].

### Inclusion and exclusion criteria

#### Inclusion criteria for infants

Inborn and outborn preterm infants of ≤ 32 + 0 weeks of gestational age (GA) and/or birthweight ≤ 1500 g will be included in the study after legal guardian written informed consent.

#### Exclusion criteria for infants

Exclusion criteria are severe congenital anomalies (e.g. cyanotic heart disease, severe lung hypoplasia, congenital diaphragmatic hernia), decision not to provide full life support, decision for palliative care before study entry, or parents with severe psychiatric disease.

While the intervention includes all infants admitted to the neonatal unit, only those infants meeting inclusion criteria and not meeting exclusion criteria are eligible for the study.

#### Inclusion and exclusion criteria for staff

Inclusion criteria for medical staff is being a registered member of the medical team (doctor, nurse) at the neonatology department of the University Hospital Giessen, Giessen, Germany. Exclusion criteria are decline of informed consent.

The baseline cohort will consist of 45 preterm infants and their parents. Subsequent cohorts will consist of all included infants who will be recruited consecutively over 6-month-periods together with their parents. The expected number of eligible patients is 90 patients/year based on average admission during the last 5 years (2016–2020).

### Process of informed consent

All parents who’s preterm infant are meeting the inclusion criteria will be approached. Legal guardians will be asked for informed consent by good clinical practice (GCP) qualified staff members.

The study was designed in accordance with the declaration of Helsinki and approved by the Ethics Committee of Justus Liebig University, Giessen, AZ 153/20. The protocol has been registered at clinicaltrials.gov (No NCT05286983).

### Setting

The neonatal department of the University Hospital Giessen is a tertiary care 10 beds NICU together with a 27 beds step-down unit on the same floor. The step-down unit includes 8 rooming-in beds designed as double rooms with an accompanying bathroom (2 mother or father-infant- dyads per room).

### First step towards establishment of FCC

In 2017/2018 the Family Infant Neurodevelopmental Education (FINE) program was introduced as a first step to enhance FCC in the unit. The FINE programme is designed to provide a theoretical framework and practical skills to neonatal professionals based on three principles: neuroprotection, relationship and individualized care [22, 23].

### Forming the focus group

A multidisciplinary focus group including staff nurses and physicians with a special interest in FCC will be established to guide the whole project of enhancing FCC in the NICU, as a next step a member of the psychosocial support team will be included in the focus group. Including parents in FCC focus groups has been suggested to be beneficial [24] and is one of the future goals.

The focus group will meet regularly and will decide on FCC interventions as potentially better practices (PBPs). The PBPs will cover the four pillars introduced by FiCare: parent education, staff training, NICU environment and psychosocial support [13]. The team will then disseminate the changes into the greater team through workshops, hands-on teaching, displays, etc. Potential PBPs for our department enclose: Implementation of a parent education program, parent participation on rounds, parent presence during handover, parent skill self-assessment, systematic parent-to-parent support, implementation of a parent advisory board, participation of parents in the focus group, implementation of the CO- PARTNER tool to enhance and measure parent participation and collaboration with the medical team, [25] implementation of regular staff education, improvement of psychosocial support, improvement of neonatal unit surroundings to promote parent-infant-closeness.

### QI Method for Implementation

The Model for Improvement and the PDSA (Plan- Do- Study- Act) cycle will be used as QI Method to improve FCC in our level III NICU as it allows rapid tests of change and improvement of outcomes [16].

Every 6-months period 1–3 new PBPs will be introduced by the FCC team and evaluated using 2–3 repetitive PDSA cycles. Although PDSA cycle time intervals of 4 weeks or shorter have been recommended to track change in a “live system”, slightly longer time intervals were chosen as the implementation of change in a NICU setting with working in shifts slows down dissemination of change [18].

### Degree of FCC

Two different tools will be used to assess the extent of FCC implementation. The first tool asses the implementation of FCC on an organizational level and was designed by the Institute for Family Centred Care: The Self-assessment inventory of family centred newborn care [26], it was shortened and translated into German language following the guidelines for translation and cultural adaptation to assess FCC in general [27].

The second tool has been published by the FINE group [28] and focuses on the operational implementation of FCC on a daily basis.

### Infant outcomes

The primary outcome of the present study is the length of hospital stay measured by corrected gestational age (cGA) at discharge.

The secondary outcomes are presented in Table 1.

**Table 1:** Secondary outcomes

Neonatal morbidities	Bronchopulmonary dysplasia as defined by Walsh [29] Intraventricular hemorrhage ≥ Grade 3 and cystic periventricular leukomalacia as diagnosed by ultrasound [30] NEC ≥ Stage 2 [31] ROP ≥ Stage 3 or treatment of ROP
Growth	Weight gain (g/kg/d) from admission to 36 + 0/40 + 0 weeks (cGA) Z- Scores for weight, length and head circumference at 36 + 0 weeks, 40 + 0 weeks, and 3, 12 and 24 months (cGA)
Nutrition	Day of Life (DOL) of achievement of full enteral feeds defined as 150 ml/kg/d for 3 consecutive days Removal of iv access (DOL) Removal of nasogastric tube (cGA) Breast milk nutrition (Percentage of breast milk within DOL 1–14, DOL with first breast milk feed, DOL with full breast milk feed, breast milk proportion at discharge, fully breastfed at discharge
Respiratory support	End of Continuous Positive Airway Pressure (CPAP) End of Highflow Nasal Cannula (HFNC) End of oxygen supplementation
GA at start of rooming- in	
Neurodevelopmental long-term outcome	Neuropsychological Developmental Screening (Neuropsychologische Entwicklungsscreening, NES, 2005) at 12 months cGA Bayley Scales of Infant Development, 3^rd^ Edition, German Version, at 24 months of cGA Gross Motor Function Classification System (GMFCS)

### Parental outcomes

#### Parental mental health

Depression and anxiety scores of mothers and fathers will be evaluated using standardized questionnaires within the first 10 days after birth, after 4 weeks, at discharge, at 3, 12 and 24 months (corrected age of the preterm infant [32]. Depression and Anxiety score is evaluated with the German version of the Hospital and Anxiety and Depression Scale (HADS-D). The HADS-D is a validated self-report questionnaire divided into 2 sub-scales (anxiety and depression HADS sub-scales) designed to measure recent depression and anxiety symptoms and has been used for evaluation of NICU parents with a cut-off score of 8 or above for both subscales indicating clinically significant anxious or depressive symptomatology [33, 34].

For evaluation of parenting stress, the Parenting Stress Index (PSI), German Version (Eltern-Belastungs-Inventar, EBI) will be used at 3, 12 and 24 months (corrected age). The PSI has been used to evaluate parenting stress in mothers of very preterm infants up to the age of 24 months corrected age [35, 36].

#### Parental satisfaction with care and parental skills

For the evaluation of parental satisfaction with care, a questionnaire has been designed covering relevant areas such as NICU surroundings, visiting hours, medical care, or communication.

Parental skills will be parent self-assessed with a newly developed questionnaire focussing on discharge readiness and competence at home. Parental satisfaction with care and parental competences are important components of FCC.

#### Parent-infant-interaction

Charts and tables at the bedside will be used to assess the degree of parental involvement in the care of the infant (e.g. feeding, bathing, etc.) during the hospital stay and to be able to analyse whether parental involvement increased during the intervention.

Parents being the primary caregiver of their infant are at the core of FCC [6]. This includes being actively involved in the nursing care from admission onward, being involved in decision making and being able to visit the infant unrestrictedly.

#### Parental visiting hours

Visit duration and time spent kangarooing will be recorded separately for mothers and fathers.

### Staff outcome

Satisfaction of the medical team with the work environment will be evaluated using part of the German version of the Copenhagen Psychosocial Questionnaire (COPSOQ). The COPSOQ is a valid and reliable tool for workplace satisfaction surveys [37]. Workplace satisfaction will be evaluated every 6 months.

### Timeline

The current status (baseline data) will be assessed in a baseline cohort of 45 preterm infants and their parents, as this is the average number of patient admissions per 6 months fulfilling the inclusion but not meeting the exclusion criteria during the last 5 years. The following cohorts will consist of all included infants who are recruited consecutively within 6-month-periods and their parents. The duration of the study will be 5.5 years with the first interim analysis after 2 years of start of the first intervention phase. A study period of 5.5 years for recruitment was chosen because shorter time periods have been associated with lacking results [7].

### Statistical assessment

**Fig. 3:**
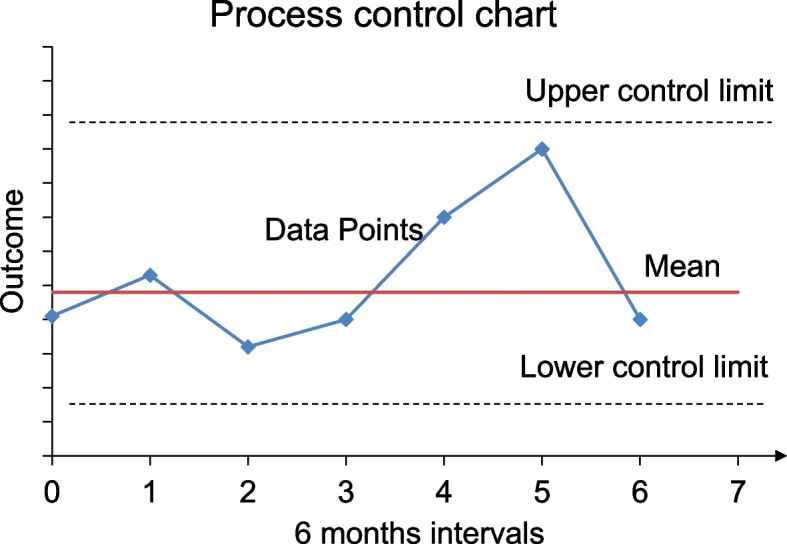
Process control chart

Trend analyses and process- control charts (Sample Process Control Chart, Fig. 3) will be used for outcome assessment as used previously in quality-improvement studies [38].

Process control charts will be used to describe, visualize, detect and understand changes in processes of care and outcomes [39]. Crude rates or medians will be plotted using 6-month periods to provide sufficient time points for process control chart analysis while maintaining more than 40 observations for subgroups [39]. For each graph, upper and lower control limits will be determined using standard statistical process-control parameters (± 3 standard deviations) using 6 months baseline data. We aimed to have 21 data points. Special-cause variation in outcomes will be defined as (significant changes that are not part of random variation) as any data point beyond the control limits, or 6 sequential points on 1 side of the mean, in either direction [38].

Second, linear trends for changes in FCC care practices, patient characteristics, and outcomes will be analyzed using F or Wald χ2 tests from linear or logistic regression models with 6 months periods used as continuous variable. Models will be adjusted using a generalized estimating equation approach (to account for clustering) with symmetric covariance structure, for the common variables known to be associated with the outcomes: e.g. gestational age in weeks (as a categorical variable), sex, demographics, multiple births, small for gestational age, outborn status, Score for Neonatal Acute Physiology version II (SNAP II) greater than 20, antenatal steroids, and change in major medical interventions. Change in major medical interventions was included because there may be future changes such as general stem cell transplantation or artificial placenta, which may have a tremendous effect on the long term outcome.

Data management and statistical analyses will be done using Microsoft^R^ EXCEL and R version 3.2.2 A 2-sided p value of less than 0.05 will be regarded as statistically significant. Data imputation will be done by study nurses and students and cross checked by the GCP qualified investigators. Source data will remain within the study site.

### Sample size calculation

#### Primary outcome: corrected GA at discharge

A sample size of approximately 110 parent/child dyads is required to achieve a power of 95% in a single factor analysis of variance with cGA as dependent variable and implementation status of Fine + criteria as independent variable with a global significance level of 5% when comparing 11 implementation levels/time points to prove a reduction in cGA at discharge of two weeks. The expected number of at least 300 enrolled infants (very conservative estimation) will be sufficient to prove the targeted reduction in cGA and to compensate for unknown parental study compliance and unexpected drop-outs.

##### Background

In our department in 2018–19 in VLBW infants the mean GA was 29.0 ± 2.9 weeks. The mean cGA at discharge was 38.9 ± 4.0 weeks. Five infants were discharged beyond 45 weeks of cGA due to severe clinical complications. These infants were excluded from sample size estimation. In the remaining VLBW infants the mean GA at birth was 29.2 ± 2.8 weeks and the mean cGA at discharge was 38.3 ± 2.1 wks. In the subgroup of infants of 27 to 31 + 6/7 weeks the mean GA was 29.3 ± 1.3 weeks and mean the cGA age at discharge was 37.8 ± 1.6 weeks. In a recent paper, the cGA at discharge was 34.7 weeks in 52 infants 27–31 weeks of gestation [15]. Therefore, reduction in mean cGA at discharge by two weeks (estimated effect size 0.5–1.1) is a realistic and achievable target.

## Discussion

The current project aims to enhance FCC in a level III NICU in Gießen, Germany. The FINE (Family Infant Neurodevelopmental Education) education in FCC has been introduced as baseline but FCC depends on repetitive evaluation, propagation of its aims and stepwise implementation of further focus fields. The study continuously assesses whether the implemented changes to advance FCC improve FCC indicators and multidimensional outcomes. Infant, parents and staff outcomes are evaluated separately and interactions between these three dimensions will be analyzed as well. To our best knowledge, this is the first study systematically assessing the further enhancement of FCC in such a comprehensive way. A particular strength is that all data will be recorded separately for mothers and fathers. Although fathers have become more involved in the care of their preterm children only few studies have focused on the visiting patterns, involvement and well-being of fathers [40–43]. This study will provide further insight and can help to better support fathers of preterm infants.

Data on measurable indicators showing an increase of parental participation in the care of their preterm infant as a result of FCC interventions is scarce [43]. The standardized assessment tool and the CO-PARTNER tool will thoroughly document parental participation in several areas (kangarooing, visit time, different caregiving activities like bottle feed/breastfeeding, diaper change…) through charts at the bedside to be able to assess whether FCC interventions have an impact on parental skills.

Several professional societies endorse FCC by policy statements [1]. Although these policy statements exist, the practical implementation is varying across different countries, but also within countries [4, 5]. Disseminating innovations in health care is challenging with a considerable gap between knowledge and practice. To make change happen it is not enough that evidence for an intervention is available. The more difficult part is to translate evidence into local clinical practice [44]. To facilitate positive change management, a multidisciplinary team including nurses and physicians was implemented in the first stage with the aim to complement the team with parents of former preterm infants. This team consists of staff members with a special interest or expertise in FCC and will lead the process of innovation adjusted to the local neonatology department [45]. A multidisciplinary focus group was chosen to guide the implementation as successful dissemination of innovations in health care that is best achieved if a clinical guideline or recommendation is not merely repeated, but reinvented by the team and adjusted to the local context [19, 44]. The EPICE (Effective Perinatal Intensive Care in Europe) study group has identified the following three crucial factors for making change in the NICU happen: Innovation ideas that come from the NICU team itself and not from outside, active participation of the unit staff in the development and implementation of the innovation or policy change and the presence of staff members with personal interest in making the change happen [45].

FCC has been described as a journey with different neonatal units at different stages [46]. We have started on this journey four years ago and want to move further along the way.

QI efforts in healthcare have demonstrated variable results, some reporting positive changes in patient outcomes, others reporting no changes [20, 47, 48]. One possible reason for this variability is that e.g. the PDSA cycle has not been implemented rigorously in many studies [17, 18]. If implemented well, the PDSA cycle has the potential to lead to sustained improvement in healthcare and enable stepwise assessments and timely intervention [20]. Therefore, it is reasonable to not discard the method as such, but diligently apply the method. We will thoroughly consider the five key elements of the PDSA cycle identified as crucial by Taylor and Knudsen [17, 18]. The reporting will be done according to the SQUIRE 2.0 publication guidelines to ensure completeness, precision and transparency [49].

Changing the culture of a unit and the attitudes of individuals is challenging [7] and it is a very sensitive process. Acknowledging this Quality Improvement (QI) project as such a sensitive process we will evaluate staff satisfaction regularly. The implementation of changes often leads to an initial decline in staff satisfaction, followed by a recovery [50]. The scheduled 6 monthly assessment of staff satisfaction will enable us to realize early whether after an expected initial decline staff satisfaction recovers again.

This study will provide important insight into the application of a QI method to advance FCC in a NICU regarding the success of the QI method in achieving a higher degree of FCC and regarding team satisfaction during this change process.

Moreover statistical process control methods will be used as a novel approach to prospectively analyse the data over time and to understand and address the performance of the QI project [39]. This technique enables clinicians to detect process changes and trends earlier with the help of graphical display [51]. This approach has been suggested as a beneficial tool in neonatology [39, 47, 52]. The Canadian Neonatal Network recently published the results of a quality improvement initiative after a period of 14 years of experience using this technique [38]. Naturally, clinical data has some variation over time that is independent of interventions. Process control charts help to distinguish between natural variation and variation due to specific interventions.

The COVID pandemic starting at the beginning of our study was a big challenge as access to hospitals was greatly restricted in Germany also for the parents of NICU patients. Our results might give further insight into the consequences of these restrictions for the infant, parents and staff.

There are as well limitations to our study. The study is a single centre study undertaken in one neonatology department in Germany. The context of the particular neonatology department involved and its patient demographics including the socioeconomic and cultural background will influence the results. Due to the single center design the number of patients is restricted and a recruitment period of 5.5 years is needed. Changes of e.g. nurse-patient ratios on the basis of modifications of the obligatory national guideline of the Joint National Committee of Germany or towards the provision of active support according to the national AWMF guideline recommendations for the care of preterm infants at the border of viability will have an impact on the study results. Possible demographic changes over the study duration will also affect results. Nevertheless, our study pioneers the comprehensive evaluation of infant, parental and staff outcomes and when implemented successfully, the studied comprehensive assessment tool can be used to monitor and guide FCC interventions within a local or multicentre context in any other NICU.

## Data Availability

Not applicable.

## Abbreviations

FCC: Family Centered Care
NICU: Neonatal Intensive Care Unit
PDSA: Plan- Do- Study- Act
FiCare: Family Integrated Care
NIDCAP: Newborn Individualized Developmental Care and Assessment Program
GA: Gestational Age
GCP: Good Clinical Practice
FINE: Family Infant Neurodevelopmental Education
PBP: Potentially Better Practices
DOL: Day of Life
CPAP: Continuous Positive Airway Pressure
HFNC: Highflow Nasal Cannula
NES: Neuropsychologische Entwicklungsscreening
GMFCS: Gross Motor Function Classification System
HADS-D: Hospital and Anxiety and Depression
PSI: Parenting Stress Index
EBI: Eltern-Belastungs-Inventar, EBI
COPSOQ: Copenhagen Psychosocial Questionnaire
cGA: Corrected gestational age
EPICE: Effective Perinatal intensive Care in Europe
QI: Quality Improvement
FINE: Family Infant Neurodevelopmental Education
NEC: Necrotizing enterocolitis
ROP: Retinopathy of prematurity
